# Supplementary material for: Are carbon emissions trading and green financial instruments synergistic? -Comprehensive quantitative research based on content analysis
Source: PLoS One. 2024 Mar 7;19(3):e0298601. doi: 10.1371/journal.pone.0298601 (PMC10919601; doi:10.1371/journal.pone.0298601)
Supplement: S1 Data — (ZIP) [file pone.0298601.s001.zip › Data/地级市Z_F工作报告.zip!/╔╜╬≈/╘╦│╟╩╨/└┤╫╘╣1⁄2╓┌║┼í╛┬φ┐╦╩2╛▌═°í┐_2014.docx]

各位代表:

现在，我代表市人民政府向大会报告工作，请予审议，并请市政协委员和其他列席人员提出意见。

一、2013年工作回顾

2013年是运城转型跨越发展踏上新起点的一年。面对复杂的宏观经济形势，全市人民深入贯彻落实党的十八大和十八届三中全会精神，紧紧围绕“五大战略重点”和建设“美丽河东、大美运城”的美好愿景，创新发展理念，拓宽转型路径，点燃工作激情，狠抓工作落实，全市经济发展活力增强，社会保持和谐稳定。

全市地区生产总值完成1140.1亿元，增长9.2%；规模以上工业增加值完成406.3亿元，增长13.1%；固定资产投资完成1008.9亿元，增长22%；社会消费品零售总额完成552.1亿元，增长14.3%；外贸进出口总额完成17.5亿美元，增长64%；财政总收入完成91.2亿元，增长13.9%；公共财政预算收入完成45.4亿元，增长9.3%；城镇居民人均可支配收入完成20718元，增长10.6%；农民人均纯收入完成7198元，增长12.8%。省政府考核的9项主要经济指标中，我市8项指标增速前移在2-10个位次之间，其中财政总收入、规模以上工业增加值、外贸进出口总额3项指标增速全省第一，固定资产投资虽然没有进位，但超额完成目标程度在全省排名第一。

一年来，我们紧紧围绕“五大战略重点”，开阔视野，深入调查，认真研究，进一步明晰了发展思路和推进路径工业新型化方面，按照“全市经济一盘棋，错位发展、差异竞争、优势互动”的思路，坚持传统产业改造提升和新兴产业园区化发展集群化招商两条路径，大力发展产业集群。农业现代化方面，按照“土地规模化、组织企业化、技术现代化、经营市场化”的思路，推动土地流转、农业产业板块设计招商、农产品深加工和农产品物流体系建设。市域城镇化方面，按照“工业化、信息化、城镇化、农业现代化和城乡生态化”互动推进的思路，探索“工业集群化、集群园区化、园区社区化、社区城镇化、土地规模化、城乡生态化”的互动机制，努力构建有运城特色的统筹城乡一体化发展之路。在总体布局上，统筹中心城市、大县城、小城镇、新农村功能互补、协调发展；在城市建设上，突出“城市功能完善、产业空间拓展、土地集约利用、市民方便宜居”四个目标一个过程实现。城乡生态化方面 ，生态绿化按照“山上治本、身边增绿、林业增效、产业富民”的思路，着力提高森林覆盖率；城市绿化做到“横有厚度、纵有层次，点成缀、线成景、片成林”；环境保护坚持“整治修复并举、治本治源结合”，强化节能减排，淘汰落后产能，加强大气、水、土壤污染防治，持续改善生态环境。文化旅游产业方面，按照“高端创意、整合资源、政府引导、市场运作”的思路，加快文化与旅游深度融合。

一年来，我们按照这样的思路和路径，科学做好总体布局，强力推进工作落实，“五大战略重点”取得了令人振奋的成绩工业发展势头喜人。工业增速和效益名列全省前茅。全市规模以上工业增加值增长13.1%，增幅比全国、全省分别高3.4个和2.6个百分点，实现利税98.6亿元，增长48%；中小企业完成总产值1296.8亿元，增长13.7%。全市工业增加值和实现利税两项指标增幅全省排名第一，是近年来工业发展最好的一年。园区化集群化发展成效初显。根据既有的产业基础和比较优势，全市13个县(市、区)和5个开发区精准定位了32个主攻产业和52个专业化链条。进入工业园区的项目167个，占到项目总数的68%。”5+15″工业园区累计完成工业总产值1100亿元，占全市工业总产值的67%，成为全市工业发展的主战场。

产业集中度进一步增强。产业集群规划项目投资1131亿元，占工业重点项目总投资的78%，园区新签约和开工建设的项目80%以上都同产业集群密切关联。传统产业改造提升步伐加快。围绕煤电铝材、煤焦化和金属镁等产业链条延伸，推动传统产业改造升级。铝深加工产量40万吨，占到全省的87%。煤矿复工复产数达到14座，煤炭产量达到855万吨。与省内外大企业大集团联合重组步伐加快，蒲州电厂、河津电厂、永济电厂与同煤集团实现了联合重组，大唐运城电厂与陕煤集团成功合作，阳煤丰喜集团与山西海丰铝业达成产权置换协议。焦化行业整合重组进展顺利。实施创新驱动战略，新增4家省级企业技术中心。产学研合作进一步深化，被确定为“山西省产学研合作示范基地”。金融支持实体经济力度加大。全年贷款余额净增125亿元。银保企洽谈会协议信贷总额696.3亿元，履约率达97%以上。

金融业实现税收3.5亿元，增长57%。“运城信贷融资服务平台”顺利开通。荣获“中国金融生态示范城市”称号。

“三农”工作全面提升。全年粮食产量31亿公斤，水果总产量53亿公斤，双双再创历史新高。水果出口7000万公斤，增长133%。肉类和禽蛋总产量分别增长5.5%、5.9%。农村土地流转力度加大。在全省率先出台了《关于积极引导农村土地流转促进农业现代化发展的若干意见》。市财政列支2740多万元对农村土地流转进行奖补，列支500万元作为土地流转市级风险储备基金。新绛、盐湖土地流转的经验得到省委、省政府的充分肯定。全市土地流转面积达到123万亩，位居全省第一。农产品加工业增势强劲。农产品加工销售收入达到205.4亿元，增长18.7%，已占到全省的1/5。农村基础设施建设不断加强。完成农村公路建设改造873公里。小浪底引水、禹门口灌区东扩、北赵引黄二期等重点水利项目开工建设。新建和改造末级渠道1621.3公里，新增水地面积25.6万亩。“一村一品”专业村累计达到949个，占行政村总数近30%。专业村主导产业人均收入达到4530元，占到农民人均纯收入的65%。新增农民合作社1920家，累计达到8755家。现代农业示范区建设力度加大。7个省级现代农业示范县累计建设各类示范园区235个，完成投资77.9亿元。

城镇化步伐不断加快。强化规划引领，挖掘城市价值。编制完成了10余项城市专项规划、12个县(市)总规、6个重点镇近期建设规划和20个乡镇总体规划，中心城区控规覆盖率达到53%。对东部新区、高铁商务区等重点片区重新进行功能定位与城市设计。中心城市、大县城、小城镇、新农村四位一体统筹推进。实施城镇化项目215项，完成投资103.9亿元，城市建设力度是近年来最大的一年。中心城市坚持路、水、林、产四管齐下，统筹推进“八区联动”，辐射带动能力得到新提升。禹都公园、高铁站前广场、工农街跨解放路高架桥等54项重点工程顺利推进，完成投资57.7亿元。新修道路74.2公里，11条城市主干道全部打通。运城经济开发区、空港经济开发区区域主干路网、配套设施进一步完善，产业集聚步伐加快。大县城实施城镇化项目161个，完成投资46.3亿元。”5+15″工业园区和开发区在新一轮规划中，全部设计了城市社区，有的同步开展了社区建设，成为通过产业集聚带动人口集聚，推动城镇化的重地。15个重点镇“五建设两整治”共完成投资8.6亿元。乡镇商贸中心和农村物流配送中心规模化发展，农民消费更加便捷、实惠、安全。330个新农村重点村“四化四改”和“五个一工程”全面完成。大力实施13个连片示范区建设，完成投资8.2亿元。生态智慧城加快建设。绿坡、治湖、兴业全面展开，完成了《生态智慧城概念规划》和《盐湖生态文化旅游风景区概念规划》，进一步明确了产业定位。盐湖周边环境整治、绿化造林和湖面扩大等工作积极推进。盐湖生态文化旅游景区、碧桂园商业街及高端居住社区等一批重大项目达成投资意向。城市管理水平不断提升。扎实推进“四城联创”，集中开展交通秩序、非法营运三轮车及环境卫生综合整治，市容市貌明显改观。

城乡生态持续改善。造林绿化成效明显。全年造林40.4万亩，森林覆盖率提高1个百分点。发展以核桃为主的干果经济林20万亩。平陆县被评为“全省林业生态县”。创建国家生态乡镇1个，省级生态县1 个、生态乡镇36个、生态村192个。生态水系工程建设稳步推进。以“一池、四库、三渠、一河、四滩”及各公园水系贯通为主的城市水系修复项目启动实施，涑水河、汾河环境综合整治扎实推进。节能减排力度加大。淘汰铁合金、造纸、印染、电力等行业落后产能企业6家。大力发展循环经济，工业固体废弃物综合利用率达到67.5%。全市万元GDP综合能耗下降3.82%，化学需氧量、氨氮、二氧化硫、氮氧化物、烟尘和工业粉尘等6项主要污染物年度减排任务全面完成。

文化旅游产业快速发展。制定了《运城市加快文化旅游产业发展若干意见》，重点实施盐池生态圈保护与修复、关圣文化建筑群建设、文化创意园区培育、青铜文化产业壮大、包装彩印文化园提升和历史文化古城保护六大工程。《关圣文化建筑群申遗工作实施方案》编制完成。成功举办关帝圣像巡游台湾和福建活动，关公文化影响力进一步扩大。文化产业开发力度加大。开工建设文化项目15个，累计投资50.8亿元。参加深圳文博会和山西首届文博会取得丰硕成果，共签约项目16个，达成合作意向228亿元。赴北京、上海、杭州、南京、广州等地深入开展旅游宣传推介，开通了重点城市旅游专列，运城机场进出港人数首次突破100万人次。全年旅游总收入212.6亿元，同比增长28.1%。一年来，我们克服财力紧张的困难，着力保障和改善民生，人民生活水平得到新提高越是财力紧张，越要首先保障民生，确保财政资金向民生倾斜。全年拿出巨额资金，弥补民生方面的历史欠账。全市民生支出186.3亿元，占到财政总支出的81.2%，是民生支出总量最多的一年。社会事业全面发展。全市财政新增1000万元扶持教育事业发展，并动员社会各界积极参与，资助了2240名家庭经济困难的大学生顺利入学。加强教师队伍建设，首次评选50名优秀教师享受运城名师待遇，清理33名不称职的中小学教师。新(改、扩)建97所公办幼儿园，学前三年毛入园率达到88.8%。新型农村合作医疗参合率达到99.6%。城乡居民人均医疗保险财政补贴由240元提高到了280元。城乡居民最低生活保障标准每人每月分别提高30元和24元，惠及27.2万人。建成农村老年人日间照料中心447个，服务1.5万余名农村老年人。转移农村劳动力13.1万人。城镇新增就业5.9万人，城镇登记失业率控制在1.54%。新开工各类保障性住房21897套，基本建成15170套。高度关切群众冷暖，每人取暖费在提高一倍之后，又增加了1000元的补助，并对采取区域锅炉、燃气锅炉等方式供热的小区或企业进行了奖励性补贴，圆满完成128万户134万吨“爱心煤”发放任务，有效保证了居民温暖过冬。“戏曲惠民、欢乐百姓”系列活动丰富了群众文化生活。25个项目通过第四批省级非遗名录审批，新增省级非物质文化遗产24项。国家级文物保护单位达到90家。群众体育、竞技体育和体育产业协同推进，成绩显著。农村五件实事进展顺利。全面完成11430户农村危房改造任务；特困群众易地搬迁项目首次扩大到8个非扶贫重点县；1928个行政村街道亮化任务圆满完成；改扩建69所村级幼儿园；乡村清洁工程取得阶段性成效。安全生产持续稳定好转。深入开展安全生产大检查和食品安全专项检查，加强重点领域安全监管，全年各类生产经营性事故起数和死亡人数又有新的下降。

一年来，我们坚持以开放促改革，以改革促发展，经济社会发展活力显著增强各项改革继续深化。行政审批制度改革深入推进。久拖未决的山西农药厂、永济纺织厂破产清算问题取得突破性进展，由省财政支付的破产费用基本落实。运城大酒店、市建筑工程有限公司改制工作基本完成。在全省率先将13个县(市、区)公立医院全部列入省改革试点，全省医改工作现场会在我市召开。公交改制平稳实施，建立了“国有主导、多方参与、公司经营、规模发展”的运营新机制。转型综改全面推进。“四化同步”、“五规合一”等改革深入实施。理顺了省级开发区与所在县(市、区)在土地利用、行政管理和财税分配等方面的关系，明确了开发区的产业定位，极大地激发和释放了开发区的发展活力。招商引资实现新突破。拓宽开放视野，增强开放胆识，创新开放路径，坚持“小分队”走出去、“大部队”引进来，大力开展专业化招商、定向化招商、集群化招商、产业链招商，全年招商引资到位资金691.2亿元，同比增长22.9%，超额完成省定目标任务的140%。全市10亿元以上续建和新开工项目，由年初的63项增加到年底的101项，项目总投资规模大幅增长。以全省“飞地经济”试点市为契机，加大招商引资力度，引进了一批转型跨越发展的大项目、好项目。义乌国际商贸城综合工业园已经建成，投资600亿的深圳家居产业园成功签约并将落地开工。对外交流合作不断扩大。《晋陕豫黄河金三角区域合作规划》上报国务院，即将上升为国家战略。与杭州市、伊春市、黑河市成为友好城市，拓宽了我市与经济发达地区和边贸旅游城市的交流合作空间。积极推进大通关建设，运城海关、出入境检验检疫大楼主体完工，成为我市进一步扩大开放的重要标志。全市新增外商投资企业7家，新增对外贸易经营企业30家，进出口国别或地区达到121个。合同利用外资7715万美元 。

一年来，我们坚持依法行政，改进社会治理方式，民主法制和精神文明建设进一步加强自觉接受人大法律监督、政协民主监督和社会舆论监督，认真听取各民主党派、工商联和无党派人士意见。全年办理人大代表建议意见366件、政协提案179件。精神文明创建活动扎实推进。“六五”普法深入开展。认真贯彻党的民族宗教政策，民族团结局面进一步巩固。加强和改进社会治理，强化信访和矛盾排查调处工作，社会保持和谐稳定。双拥工作进一步加强，军民融合式发展深入推进。计生、审计、统计、档案、地震、文物、人防、地方志、外事侨务等工作不断加强，妇女儿童、老龄、残疾人等各项社会事业也取得了新的成绩。

各位代表，回顾一年来的工作实践，我们深切的体会到：必须以开放的视角审视我们的优势，必须以创新的智慧整合我们的优势，必须以发展的自信彰显我们的优势。一年来，我们理清了思路，明确了路径，收获了果实。这些成绩的取得，是贯彻落实党中央、国务院和省委、省政府一系列决策部署的结果，是市委统揽全局、坚强领导的结果，是市人大、市政协倾力支持、有效监督的结果，是全市人民辛勤工作、团结拼搏的结果。在此，我代表市人民政府，向全市各条战线的广大干部群众，向大力支持政府工作的人大代表、政协委员、各民主党派、工商联、无党派人士和人民团体，向驻运部队、武警官兵和公安干警，向所有关心支持运城发展的社会各界人士，表示衷心的感谢和崇高的敬意!

各位代表，成绩已成过去。走向新的一年，我们也清醒地认识到，运城经济社会发展中还存在着不少深层次的矛盾和问题：工业经济总量偏小，布局分散；农业加工转化率低，现代化水平不高；文化旅游产业虽然资源丰富，但整体策划和产业开发程度不够；城镇化率低于全国全省平均水平；社会事业欠账较多，民生改善任务较重；政府职能转变滞后，政务环境有待进一步优化。对这些问题，我们一定要本着对运城人民高度负责的精神，采取有效措施，努力加以解决，决不辜负人民的厚望和重托!二、2014年工作安排

2014年是我们推进“五大战略重点”深入实施，为运城新一轮经济社会发展夯实基础的重要一年。今年政府工作的总体要求是：深入贯彻落实党的十八大、十八届三中全会、习近平总书记一系列重要讲话精神和全国、全省“两会”、市委三届五次全会精神，坚持稳中求进、改革创新的总要求，以转型综改试验区和晋陕豫黄河金三角承接产业转移示范区建设为统领，以开展党的群众路线教育实践活动为契机，全面深化改革，强化创新驱动，统筹推进工业新型化、农业现代化、市域城镇化、城乡生态化和文化旅游产业“五大战略重点”，大力推行“核心在转型、重点在项目、关键在领导、根本在落实”的工作机制，为建设美丽河东、大美运城努力奋斗。

2014年全市经济社会发展的主要预期指标是：地区生产总值增长9%左右，规模以上工业增加值增长12.5%，固定资产投资增长20%，社会消费品零售总额增长12%，财政总收入增长10%，公共财政预算收入增长8%，城镇居民人均可支配收入增长11%，农民人均纯收入增长13%，城镇新增就业岗位5.65万个，城镇登记失业率控制在4%以内，居民消费价格水平涨幅控制在3.6%左右。

约束性指标是：万元地区生产总值综合能耗、万元工业增加值用水量和化学需氧量、氨氮、氮氧化物减排完成省下达任务，万元地区生产总值二氧化碳排放量下降3.5%，二氧化硫下降2.96%，烟尘、粉尘排放量分别下降2.28%、2.67%。

围绕上述目标任务，重点抓好以下九方面工作：

(一)全面深化改革开放，不断提升创新发展新境界

切实转变政府职能。加强对政府工作的顶层设计，推动政府部门的职能整合与互动，提高公共资源的利用效率和行政效率，推进治理能力现代化。进一步简政放权，再取消和下放一批行政审批事项，切实优化审批流程，推进“一站式”服务。加快工商登记制度改革。完成市、县政府机构改革，推进事业单位分类改革。严格控制机构编制和财政供养人员。培育中介组织，发挥其在社会事务管理中的作用。

深化国有企业改革。实施“国企改革三年推进计划”，通过联合、重组、破产等形式，一企一策，分类推进，发展壮大一批、改制退出一批、关闭破产一批、整体下放一批，利用3年时间，基本完成全市国有企业的改革改制。

深化财政体制改革。改革预算制度，实行零基预算。推行财政扶持资金的竞争性分配，提高财政资金的利用效率。加强地方政府债务管理，防范财政风险。积极探索社会资本进入城市基础设施领域新途径，推行政府购买公共服务新模式。同时，加强税收征管，严防偷税漏税，落实地方税收保障办法，推进社会综合治税。

探索创新金融支持地方经济机制。继续实行财政资金存储规模与银行放贷规模挂钩的机制，鼓励银行支持地方发展，力争新增贷款达到130亿元。鼓励金融创新，构建“企业+担保+风险补偿+奖励+银行”的融资机制，支持产业集群化发展和重点项目建设。围绕建设黄河金三角区域金融中心目标，加快推进交银大厦和鑫源大厦金融双子楼建设，着力打造东部新区金融商贸区，启动实施北部高铁商务金融 港。健全金融体系，积极引进银行、非银行金融机构入驻，加快农商行发展，鼓励民间资本设立新型农村金融组织和民营银行，力争年底再有2-3家农商行开业。加快推进企业上市，积极引进私募股权基金和产业投资基金，扩大直接融资比例。举办好第十届银保企洽谈会，力争签约总额800亿元。

深化区域合作，提升开放水平。积极推进综改转型试验区建设。加快推进晋陕豫黄河金三角区域合作规划审批工作。探索建立“黄河金三角跨区域政府合作协调机制”，牵头创意策划，争取更大发展空间。加强与杭州、伊春、黑河、北京西城区和天津南开区等友好城市的经济协作。海关、出入境检验检疫办公大楼要尽快投入运营。积极推进航空口岸建设，申请设立运城保税区。

各位代表，运城有很多优势，但我们只有用改革的胆识统领运城发展全局，以开放的眼界，把运城的资源放到更广阔的市场空间去配置，这些资源的价值才能得以彰显，这些资源的优势才能得以发挥。

我们要把运城人的聪明与智慧，更多的凝结在推动运城改革开放和创新发展上来。

(二)狠抓园区化集群化发展，加快推进工业新型化

改造提升传统产业，延伸产业链条，实现循环发展。重点抓好“煤—电—铝—材”、“煤—焦—化”和金属镁产业链。煤—电—铝—材产业链要抓住国家淘汰落后产能的机遇，整合资源，进一步提升铝行业发展水平。要坚定不移地推进铝产业链的延伸，加强技术攻关，发展高端铝产品加工，通过延伸产业链和煤、电、铝联营整合，降低成本，提高铝产业的竞争能力，加快建设全国铝加工基地。积极协调山西国际能源、中煤华晋、华泽铝电参股合作，加快建设2×35万千瓦低热值煤发电及铝深加工项目，实现煤电铝材一体化。加快永济2×35万千瓦蒲州电厂和华圣铝业的整合力度，实现铝电联营。抓好阳煤集团60万件汽车发动机铝合金缸体项目，提升铝深加工能力。复晟铝业一期9月份建成投产,二期开工建设。煤—焦—化产业链要以循环经济为路径，以产能置换为基础，以化产回收为重点，着力抓好河津阳光集团和稷山西社工业园的煤焦化产业循环经济园区，以及新绛煤化工产业循环经济示范园。金属镁产业链要以银光华盛镁业为龙头，加快项目建设进度，形成以镁合金铸件、型材、锻件、板材为主体，终端产品相匹配的完整产业体系。加快中条山有色集团10万吨金属镁项目建设。同时，对钢铁、化工等传统产业，也要加快整合力度，延伸产业链条，提高传统产业竞争力。

坚定不移地推动主攻产业园区化集群化发展。重点抓好煤电铝材、煤焦化、运输装备制造、金属镁、化工、医药、农副产品加工、新材料、家具制造等9大产业集群，这9大产业集群基本涵了”5+15″工业园区所确定的32个主攻产业。各县(市、区)、各开发区都要高度重视，全力抓好各自的主攻产业。今年，每个主攻产业的新上项目不得低于10个，落地资金不得低于30亿元，当年完成投资不低于10 亿元。力争用5年左右的时间把9大产业集群做成全省乃至全国有一定市场影响力的产业集群，经济总量占到全市工业的80%左右，成为我市转型跨越发展的重要支撑。要把园区作为产业集群发展的主阵地，强化综合服务功能配套，统筹做好园区总体规划、产业发展规划、土地总体规划、环保规划等，使各类生产要素向园区倾斜。要积极对接省内大型煤炭集团,争取他们来我市投资主攻产业集群的龙头企业。用足用好工业扶持资金。今年，市级工业扶持资金增加到2个亿，鼓励竞争发展，不搞平均分配。重点支持产业集群龙头企业、公共服务平台、共性关键技术攻关和产业链延伸，充分发挥工业扶持资 金的导向和激励作用。各县(市、区)、开发区也要建立工业扶持资金，投入园区化集群化发展，最少不低于3000万元。加大对集群发展的组织领导和考核力度，实行市级集群进退流动管理。实施产业集群部门包联责任制，明确牵头部门和配合部门，举全市之力发展产业集群。对纳入市级产业集群考核范围的32个主攻产业进行流动考核，达不到标准的，淘汰出市级集群范围，取消参与市级集群发展资金分配资格；对发展较快、达到市级标准的县级主攻产业，纳入市级产业集群范围，参与市级扶持资金分配，促进产业集群争先发展。加大金融对产业集群发展的支持力度。落实《银行业加强金融服务支持实体经济实施方案》和《金融支持产业集群化发展的意见》，引导金融机构扩大信贷投放，加大对实体经济和产业集群发展的支持力度。每家金融机构至少对接一到两个主攻产业，并建立长期、稳定、高效的银企战略合作关系，确保重点主攻产业贷款余额年均增长30%以上。

支持民营经济和中小微企业集群化发展。加快中小微企业创业基地和公共服务体系建设，实现同业聚集、相互协作，形成上下游配套的产业集群。全年孵化小微企业2000户，培育新增规模以上工业企业35户，发展壮大亿元“小巨人”企业10户。

加快信息化建设步伐。提高对信息化的认识。信息化不只是辅助的工具，而且会深刻地影响我们的思维方式、工作方式、生活方式和产业模式。要申报建设国家智慧城市；切实推进已经签约的山西省首个“宽带示范城市”建设；加强与移动、联通、电信三大运营商的合作，推进大数据平台建设；切实推动与中兴集团签约的智慧城市运行平台建设；与生态智慧城建设相一致，大力发展电子商务产业集群。政府部门要以身作则，大力推进电子政务建设，提高办公方式的现代化和行政效率。在今后的城市规划与建设中，必须像对待水、电、气、暖一样，同时规划设计建设信息基础设施。下决心发展与支撑智慧城市建设相关联的智慧产业，如电子制造、信息服务、远程教育、网上培训等。注重工业化与信息化的融合发展，重点抓好大运重卡、银光镁业试点示范企业建设。

各位代表,运城之短在工业，运城的希望也在工业，工业的希望在园区，园区的希望在集群。我们一定要坚定不移地推进园区化发展集群化招商，力争通过5年左右的努力，使园区化集群化成为经济转型跨越的新引擎，铸就未来运城工业的基本格局。

(三)高度重视“三农”工作，加快推进农业现代化

稳定发展粮食生产。重点建设60个粮食万亩高产示范片，实施中低产田改造30万亩，确保全年粮食播种面积稳定在950万亩左右，粮食总产达到23亿公斤以上,积极为全省粮食安全做贡献。加强农业基础设施建设，继续实施“三引六扩、河库成网”规划，加快小浪底引水、北赵引黄二期和国家小农水重点县项目等工程建设。加快土地整理和高标准基本农田建设。提升主要粮食作物综合机械化作业和科技服务水平。

积极引导土地流转。以提高农业劳动生产率和促进农民增收为核心，先确定土地规模经营的投资主体，后引导农民流转土地，积极稳妥地推进土地规模化经营。要以盐湖区、新绛县和省里确定的71个试点村土地经营权确权试点工作为重点，探索建立土地流转的长效机制，力争年内新增土地流转面积80万亩。大力扶持新型农业经营主体，新培育100家重点示范合作社和家庭农场。

着力构建农产品物流体系。加快大型综合农贸批发市场、物流配送中心建设，推进“农超对接”。重点抓好投资36亿元的夏县润恒现代农副产品冷链物流产业园、投资15亿元的运城晋善晋美农产品物流园区和省煤运公司在新绛投资20亿元建设的农业物流园区等项目。大力发展农产品电子商务、现代物流业态，开辟和拓展我市农产品销售的新路径。

加快发展特色农业。按照产业集群化发展的思路，与山西农业大学合作，结合每个县(市、区)的产业基础和比较优势，搞好农业产业规划和项目设计。市级层面做好全市特色农业发展总体规划，县级层面做好项目策划设计，并开展农业产业招商引资。苹果、蔬菜、红枣、核桃、樱桃、山楂等特色农业，要逐步实现区域化布局、标准化生产、规模化种植、产业化经营。因地制宜，大力发展“一村一品”、“一县一业”。抓好“菜篮子”工程，新增设施蔬菜2.5万亩，总面积发展到75万亩。继续抓好运城国家级农业科技园区和盐湖区、新绛县、永济市等国家级现代农业示范县(区)建设。要划定优质农产品保护区，制定严格的保护制度，提升我市农产品在全国的知名度和市场占有率。

大力发展规模养殖业。充分利用黄河滩涂、沿山坡地和大田农业等资源优势，加强规划引导，大力发展规模养殖，力争在全市新建规模养殖场(小区)60个，弥补我市畜牧养殖发展不足这个短板。加快芮城温氏集团百万头生猪养殖、稷山晋龙集团百万只蛋鸡养殖、河津万春牧业6万头肉牛养殖、闻喜象丰农牧科技肉鸡产业化等项目建设。积极与正大集团对接，争取在临猗县投资的百万头生猪项目早日签约落地。

发展壮大农产品深加工企业。支持粟海、忠民、维之王、格瑞特、荣博辣椒等100家龙头企业做大做强，全面提升农业产业化水平，力争全市农产品加工销售收入达到221亿元，增长8%以上。

强化农产品市场营销。创新营销方式，坚持市场化导向，注重品牌建设，增强农产品的市场竞争力。要瞄准北京、上海等高端市场，精心组织运城特色农产品展销活动，邀请投资机构和国际采购商进行考察洽谈，整合农业产业链条各环节的国内外资源，带动我市农产品标准化生产与加工，不断拓宽农产品销售渠道。

加快美丽乡村建设。认真落实省政府“五件实事”部署，完成农村危房改造2万户；改造农村幼儿园40所；贫困人口易地扶贫搬迁1万人；深入推进乡村清洁工程；培训新型职业农民1万人。全年解决饮水安全人口5.3万人。加强农村生态建设。抓好扶贫开发工作，实施百企千村产业扶贫开发工程，支持革命老区建设，力争全年实现3万贫困人口脱贫。

各位代表，运城是一个传统的农业地区，我们必须通过土地规模化经营，推动农业生产组织方式的现代化；通过先进的农业技术应用，推动农业生产工艺的现代化；通过完善农业物流体系，实现农业经营的市场化，从而摆脱传统农业，实现农业现代化。(四)坚持以人为本，加快推进市域城镇化

高起点高标准抓好规划。经营城市从规划抓起。聘请高层次专家团队，对城市的总体规划、道路框架、土地利用、产业布局和生态环境保护等进行整体规划，挖掘城市价值。要维护规划的严肃性和权威性，任何人不得随意变更规划，确保一张蓝图绘到底。

“四位一体”功能互补推进市域城镇化。今年全市计划实施城建重点项目313项，总投资455亿元，年内完成投资143亿元。中心城市着眼于发挥辐射带动作用，围绕“四个100”目标，路水林产四管齐下，统筹推进“八区联动”。全年计划实施项目106个，总投资243亿元，年内完成投资62亿元。老城区以解放南路临街改造为示范，全面推进城市棚户区改造。中心区启动群众呼吁多年的河东西街延长线建设，打通城区东西走廊交通瓶颈。东部新区以禹都公园生态园林建设为引领，整体推进区域开发。北部新区抓住高铁即将开通的有利时机，加紧完善区域路网，打造高铁高端商务区。南部新区以环湖路建设为重点，加快生态智慧城建设。西部关圣景区以申遗为契机，培育龙头，壮大文化旅游产业。运城经济开发区、空港经济开发区围绕产业定位，优化投资环境，让更多符合产业定位的项目落地。

高标准规划设计建设辐射黄河金三角地区的大型会展中心。抓好10个旅游酒店、10个商务写字楼、10个商业综合体的招商与建设，打造运城地标性建筑和产业承载体。市场化改造河东会堂，建设河东剧院。支持民间资本进入基础设施、公共服务等领域。大县城着眼于通过产业集聚，实现人口集聚的功能，抓好道路、水系、供电、供气、供暖、亮化、通讯、应急排水、垃圾处理、污水处理十个方面的基础设施建设和广场、公园、公共交通、剧院、图书馆、群众艺术馆、养老院、医疗卫生、学校、保障性住房十个方面的公共服务设施建设。实施项目207个，总投资212亿元，年内完成投资81亿元。小城镇着眼于繁荣农村市场和就近提升农村公共服务水平的功能，发展特色鲜明的重点镇。要把中心镇逐渐变成为附近村农民就地提供教育、医疗、养老等服务的基地。15个重点镇建设完成投资2.6亿元，小城镇基础设施建设投资增长10%。新农村着眼于发挥推动土地规模经营和作为“一村一品”空间节点的功能，引导自然村向有条件的中心村集中，逐步推动村庄结构调整,改善农村人居环境。

加快推进生态智慧城建设。按照“绿坡、治湖、兴业”的要求，抓紧编制完成《生态智慧城总体规划》和专项规划；加快环湖南路和跨湖大道改造；推进盐湖治理、环湖主题公园、山体修复等生态系统建设；加快中央商务区会展中心、黑泥养生城、碧桂园商业街与高档社区等招商项目的开工建设。

完善城市功能，发展现代物流。我市地处晋陕豫三省交界的黄河金三角腹地，有通达的航空、密集的高速公路。今年6月大西高铁将可开通，使运城距西安只有1小时车程、距太原只有一个半小时车程，建设区域性的现代物流配送中心条件十分优越。要以高铁商务区为中心，建设辐射黄河金三角和山西全省的物流仓储配送中心，首先在文化旅游与物流配送方面发挥我市作为山西向东、向西开放的大通道和桥头堡作用。

强化城市管理。严格城市管理执法，严肃查处违规违章建筑。探索政府购买城市公共服务的新模式，率先在环卫清洁、园林绿化等方面进行试点。全力争创国家卫生城市，继续抓好以交通秩序、门牌门匾、沿街广告、环境卫生、占道经营等为主要内容的城市环境集中整治活动，使市容市貌明显改观。

各位代表，新型城镇化的核心是“以人为本”，着眼点是解决“三农”问题，着力点是工业化。我们要继续按照“工业集群化、集群园区化、园区社区化、社区城镇化、土地规模化、城乡生态化”的思路，在工业化、信息化、城镇化、农业现代化和城乡生态化良性互动中，积极探索适合运城实际的统筹城乡一体化发展之路，实现城市让人民生活更美好。

(五)大力实施生态兴市战略，加快推进城乡生态化

抓好造林绿化。重点抓好通道、城郊森林公园和荒山绿化，着力搞好沿路、沿河、沿山、沿湖生态绿化。全年完成造林40万亩，发展苗木产业10万亩，森林覆盖率增长1个百分点。抓好城市绿化工程，新增城市绿化面积243万平方米，建成区绿化覆盖率提高0.7个百分点,绿地率提高0.5个百分点。积极倡导全社会广泛开展栽植“家庭亲情林”活动，让种树成为运城每个家庭的自觉行动，成为运城人的一种时尚、一种习惯、一种情怀。

加大节能减排力度。以循环经济试点市为契机，加大淘汰落后产能力度，关小上大、扶优汰劣，提高工业废弃物处理和利用水平。搞好新能源应用、地热资源开发。

抓好大气污染防治。严格环保执法，大力整治燃煤污染、机动车污染和重点行业污染。力争2015年底，所有火电、钢铁、水泥、石化、焦炭企业及工业锅炉完成脱硫除尘设施建设与提标改造，达到新排放标准要求。推进集中供热和燃煤锅炉改环保锅炉工作。

抓好水生态保护。逐步完善全市水网体系，统筹推进中心城市水系和水体生态治理工程，赋予城市以灵动之气。实施汾河、涑水河、姚暹渠环境综合整治。各县(市、区)要加大力度，建设并运营好污水处理厂。严格落实地表水跨界断面水质考核生态补偿机制，城市集中式饮用水水源地水质达标率达到100%。

各位代表，运城的生态在山西乃至全国都是值得自豪的发展资本。在推进工业化进程中，我们要正确处理经济发展与生态保护的关系，不仅要把生态当做我们的生存环境来维护，还要把生态当做我们的发展环境来珍视，我们要有这样的信念，那就是：要让子孙后代享受到比我们更好的碧水青山。

(六)高端创意策划，整合资源优势，加快文化旅游产业发展

搞好文化旅游产业顶层创意策划。要开阔视野、高端创意，聘请一流专业团队，对我市的文化旅游产业发展进行顶层策划，促进文化旅游资源与资本、科技、人才等现代产业元素的整合、融合，不断拓展文化旅游市场。要通过总体策划和创意，找到文化资源历史积淀与现代人文化旅游消费需求的结合点，把文化资源的历史价值转化为大众消费产品，广泛吸引国内外游客。

加大景区建设和旅游产品的开发力度。突出“一核”，打造“两带”，构建“五个集聚区”，科学规划、持续推进，把关帝庙、盐湖和生态智慧城连片打造成全市文化旅游的龙头景区、全球华人朝拜关公的圣地和驰名中外的旅游目的地。抓好投资50亿元的以李家大院为核心的唐风晋韵文化旅游休闲度假区项目落地。抓好旅游大厦、东中西三个禁门、鼓楼、运城博物馆新馆等文化旅游产业项目。支持宇达集团开拓市场，做大做强。

强化旅游宣传促销。加大城市形象宣传力度。对每个景点，都要针对不同客源确定营销主题，持续不断地组织外出促销，加大与旅游公司、旅行社的对接力度。继续在南京、杭州、上海、广州、东南亚等地开展旅游促销。

大力发展大型旅游公司。坚持政府引导、市场化运作，加快文化旅游产业开发。运城机场要与海诚公司合作，吸收社会资本参与，组建高品质的旅游公司,开展专业化、市场化的文化旅游产品推介营销, 拓宽客源市场。加大对旅游企业的奖励力度，鼓励大型旅游企业为我市招徕客源，争取长三角、珠三角地区为我市输送旅游专列和旅游包机。

推进关圣文化建筑群申遗工作。按照申遗程序要求,科学编制各类规划，抓紧完善有关法规，积极与上级部门搞好对接。加大景区周边环境整治，确保申遗工作扎实推进。

各位代表，运城有丰富的历史文化资源，我们要怀着敬畏之心，在保护好这些文化资源的前提下，更加善于通过高端策划，整合创意，使更多现代产业元素与文化旅游资源深度融合，把运城的历史文化资源打造成适合现代人精神消费的文化旅游产品，发展壮大文化旅游产业。(七)加大招商引资和项目建设力度，充分发挥投资拉动作用

持之以恒招商引资。招商引资从项目书开始。各县(市、区)和开发区要根据已经完成的主攻产业的链条设计，做好招商引资项目书。按照设计好的项目，围绕主攻产业龙头企业、产业集聚地、技术人才和市场等关键点，坚持小分队走出去、大部队引进来，大力开展专业化、定向化、集群化招商，积极引进一批大项目、好项目。要发挥中介组织作用，利用行业协会的人脉资源为我市招商引资，占领主攻产业的信息制高点和人脉制高点。全力推进“飞地经济”和“总部经济”发展。力争总投资600亿元的中国运城-深圳家居产业园早日开工。积极开展“运城之友”、“回乡创业”等主题招商。确保全年招商引资到位资金680亿元，主攻产业集群招商引资到位资金占总数的30%以上，主攻产业产值年均增长20%以上。

加大项目建设力度。计划开工项目重点抓好总投资80亿元的绛县经济开发区科创中医药健康产业园、总投资65亿元的绛县莱特通用航空产业、总投资50亿元的天昊文化综合产业、总投资50亿元的美特好物流仓储、总投资35亿元的四川波鸿威斯卡特北方工业园、总投资20亿元的红星美凯龙家居广场、总投资19亿元的阳煤丰喜焦炉煤气综合利用、总投资20亿元的同煤寺塔煤矿新建矿井等项目。同时抓好总投资88亿元的太化集团河津30万吨纯碱项目前期工作。已开工项目重点抓好总投资26亿元的北方铜业多金属综合捕集回收、总投资21亿元的际华220万件高档服装及1000万双特种鞋底和新型包装材料研发生产、总投资30亿元的永济热电2×35万千瓦电厂、总投资120亿元的盐湖高铁站商业片区开发、投资20亿元的山西同誉600万支铝合金汽车轮毂二期等项目。计划竣工投产项目重点抓好总投资100亿元的大西客运专线运城段、总投资20亿元的绛县明迈特镍铬合金、总投资18亿元的华晋纺织牛仔布生产等项目。

全力抓好土地要素协调。一是调规划。在积极争取追加规划指标的同时，各县(市、区)、各开发区要提前做好各自土地总体利用规划的调整，防止项目用地因不符合土地利用总体规划而无法落地的问题。二是增减挂。今年各县(市、区)实施增减挂项目不得低于1000亩，省级开发区争取用地指标不得低于1500亩。三是列重点。将更多的项目纳入省级以上重点项目盘子，争取土地指标倾斜。四是保供地。对全市批而未供土地进行全面清理，尚未占用的要向工业园区集中调整使用。在保证项目用地的同时，高度重视土地单位面积的投资规模和投资强度,提高土地单位面积投资对GDP和财政收入的贡献率。

各位代表，运城正处在转型跨越发展的爬坡过坎阶段，投资在今天乃至今后相当长时间内都将是推动我市转型跨越发展的主要力量。投资是衡量各级政府主要领导推动发展能力的重要标志。全社会一定要牢固树立投资意识，坚定不移地抓招商、上项目、增投资，以大招商大投资推动运城大跨越大发展。

(八)加快社会事业发展，着力保障和改善民生

千方百计扩大就业。整合人社、农委、教育等资源，围绕主导产业、工业园区、产业集群、农业产业化种养加等用工需求，针对性地搞好职业技能培训，提高培训与用工需求的衔接，增强就业创业能力。完善就业服务体系，落实促进就业的各项措施，重点做好高校毕业生、复转军人及城镇就业困难群体就业工作。

切实加强社会保障。落实各项社会保障政策。进一步提高城乡最低生活保障标准，每人每月分别提高25元、22元。企业退休人员养老金提高10%。城镇居民基本医疗保险和新农合年人均财政补助标准由280元提高到320元。做好大病致贫农民的救助工作。坚持保障性住房与工业园区的社区化紧密结合，合理布局，开工建设各类保障性住房2.6万套，建成1.2万套。

全面发展社会事业。带着感情抓教育，确保我市各级各类教育稳定保持在全省的领先位置。加快发展职业教育，培养高级职业技能人才，着力构建以就业为导向的现代职业教育体系，打造具有运城特色的专业技能村(镇)，努力创建全国职业技能培训基地和人才基地。抓好中心城区中小学幼儿园建设，解决好入园难、上学难问题。加快医疗卫生事业发展。抓好北部新区综合医院及健康城建设，抓好市、县两级远程医疗系统建设。支持中医药事业发展。重视发展养老事业。稳步实施“单独两孩”政策。进一步加大科技投入，重视科技人才的引进使用。完善现代公共文化服务体系，加快市级“五馆 一院”和县级“四馆一院”建设。完成市博物馆布展，向市民开放。发展全民健身、竞技体育和体育产业。开展“文化惠民在河东”系列群众文化体育活动。加快非物质文化遗产的挖掘申报，做好保护利用。

高度重视安全生产。严格落实安全生产“两个主体”责任，切实抓好煤矿、非煤矿山、尾矿库、消防、防汛、道路交通、森林防火、危险化学品、烟花爆竹、民爆物品等领域以及学校、商场等人员密集场所的安全隐患排查治理。重视对气体泄漏、爆炸等事故的防范。严厉打击非法超限超载。加强重大危险源检测监控管理。抓好安全隐患自查自报体系平台建设与应用、安全网络化监管等工作，进一步完善隐患排查治理体系，探索建立安全预防控制体系，确保全市安全生产形势持续稳定好转。加快创建食品安全放心城市。狠抓农产品质量安全源头监管，健全食品药品安全监管体制，用最严格的监管、最严厉的处罚、最严肃的问责，坚决治理餐桌上的污染，确保广大群众舌尖上的安全。

各位代表，运城是一个人口大市、财政小市，但是无论财政多么困难，都要确保民生投入。政府欠什么债都不能欠民生的债，这是对人民的承诺，也是政府的自我激励。

(九)加强民主法制和精神文明建设，构建和谐运城

加强民主法制建设。要认真贯彻执行市人大及其常委会的决议、决定，依法向人大及其常委会报告工作，自觉接受人大的法律监督和政协的民主监督，主动接受社会和舆论监督。积极支持人民法院、检察院依法履行职责。充分发挥工会、共青团、妇联等群团组织的桥梁纽带作用。支持各民主党派、工商联、无党派人士开展工作。认真做好第十届村委会换届选举工作。深入实施“六五”普法，增强公民法制意识。

加强精神文明建设。开展“中国梦”主题宣传教育活动，积极培育和践行社会主义核心价值观。坚持正确导向，加强网络管理，提高舆论引导能力。引深“讲文明、树新风”和志愿服务活动。开展全民阅读活动。加强未成年人思想道德建设。扎实开展群众性精神文明创建和道德模范评选活动，进一步提升市民文明素质和社会文明程度。

加强和创新社会治理。大力推进基层社会服务管理体系建设，广泛调动社会各方面力量参与社会服务管理。建立健全重大决策社会稳定风险评估机制，及时排查化解社会矛盾。深化“平安运城”建设，坚持专群结合、群防群治，健全社会治安防控体系，有效应对和处置各类突发事件，确保社会和谐稳定、人民安居乐业。做好信访维稳和应急管理工作，用法治的方式破解社会治理难题。提升城乡防震减灾能力。支持国防和军队建设，深入开展双拥工作，发展人防事业，促进军民融合式发展。搞好第三次经济普查。宣传落实好民族宗教政策，做好外事侨务、对台等工作，发展妇女儿童、老龄、残疾人和红十字会等事业，重视和加强档案、史志、气象等工作。

各位代表，今年的工作思路、目标任务、工作重点和推进路径已经明确，关键是要“实干”、“干实”。市委、市政府把今年确定为“狠抓落实年”，各级政府各个部门一定要把推动经济社会发展与党的群众路线教育实践活动紧密结合起来，改进作风，凝心聚力，铆足干劲，狠抓落实。一是加强学习，增强本领抓落实。学习的能力就是与时俱进的能力，就是面对新形势、研究新情况、解决新问题的能力。各级各部门“一把手”是政府自身建设的关键。要坚持一月一次 “政府大讲堂”讲座活动，丰富各级干部驾驭市场经济的理论知识、实践知识和操作知识，增强推动科学发展的能力和水平。二是深入调研，想透说清抓落实。各级领导干部要在深入基层调查研究上下功夫，坚持问题导向，开展问题调研；注重“解剖麻雀”，吃透吃准实情，科学理性分析。要在统筹谋划上下功夫，增强战略思维和全局眼光，善于运用战略的纵坐标与全局的横坐标来统筹谋划、科学决策，提高顶层设计能力。要在想透说清干实上下功夫，做到吃透理论上的逻辑、政策上的分寸、兄弟地区的经验、本地区的实际；善于向群众说清楚干什么、为什么、怎么干；要以身作则，务实苦干，以踏石留印、抓铁有痕的作风，带领群众把思路转化为实践行动，一抓到底，抓出成效。三是严格奖惩，联动督查抓落实。认真落实政务督查、行政监察和舆论监督“三位一体”联动督查工作机制，在强化政务督查中提高行政效率，在强化行政监察中推进作风改进，在强化舆论监督中增强工作动力，形成推动各项工作落实的合力。落实首问负责制、服务承诺制、限时办结制、过错追究制，切实提高办事效率和服务水平。对主要指标、中心工作坚持重点跟踪、随机抽查和专项督查；对任务紧、时效强的工作不间断督查推进；对城市建设、重点项目、园区化发展集群化招商等重点工作，定职责、定任务、定标准、定时限，实行一月一汇总，两月一督查，一季一推进，年终总考评。四是廉洁自律，清廉从政抓落实。认真贯彻中央、省反腐倡廉的决策部署，全面落实政府系统党风廉政建设责任制和“一岗双责”。规范政府工作人员的从政行为，坚决反对“四风”，坚持用制度管人、管权、管事。坚持依法行政、科学决策，推进政务公开。加强审计监督，实现政务审计全覆盖。各级领导干部和公务人员都要严于律己，率先垂范，只有自身做到了清正廉洁，心底无私，才能做到无私无畏，敢于碰硬，有力推动工作落实。

各位代表，运城已经站在了一个新的发展起点上，运城人敢于梦想，运城人勇于创新，运城人善于实干。让我们紧密团结在以习近平同志为总书记的党中央周围，在中共运城市委的坚强领导下，坚定信心，凝心聚力，锐意进取，真抓实干，不断开创全面深化改革、加快转型跨越的新局面，努力建设“美丽河东、大美运城”!
